# WHEN SNOWBALL SAMPLING LEADS TO AN AVALANCHE OF FRAUDULENT PARTICIPANTS

**DOI:** 10.1093/geroni/igad104.1175

**Published:** 2023-12-21

**Authors:** Justine Sefcik, Zachary Hathaway, Rose Ann DiMaria-Ghalili

**Affiliations:** Drexel University College of Nursing and Health Professions, Philadelphia, Pennsylvania, United States; Drexel University, Philadelphia, Pennsylvania, United States; College of Nursing and Health Professions, Drexel University, Philadelphia, Pennsylvania, United States

## Abstract

The scientific literature has examples of how fraudulent respondents who gainfully access studies for financial incentives negatively affect the integrity of research results. However, there have been less examples shared of participant misrepresentation in qualitative research, the implications, and how to prevent deception. This session entails the presentation of a qualitative study where we believe participants misrepresented themselves during an interview for the financial incentive. For the study, we sought caregivers for individuals with a diagnosis of Alzheimer’s disease or Alzheimer’s disease related dementia with a history of chronic leg wounds. One of our recruitment efforts was through ResearchMatch. After one ResearchMatch interviewee completed the interview, he reached out to the project manager and asked if the study flyer could be shared with others that were “dealing with similar issues”. This resulted in an uptick of people being interested in study participation. Several of these participants were scheduled to complete individual interviews on one day with an experienced qualitative researcher. Clues indicating potentially fraudulent participants were vague and illogical responses to questions, and similar nonsensical responses shared across interviews. Once potential fraud was detected, interviews were paused for the following interview day so the study team could regroup and none of the interviews were used for data analysis. The lessons learned and steps for researchers to take to help reduce the number of fraudulent individuals participating in interviews will be shared. This includes two-step screening where the study staff interacts with the potential participants through methods other than over email.

